# Clinical and CT-Based Outcomes of Anterior and Posterior Syndesmotic Augmentation Using Nonabsorbable Suture Tape for Acute Syndesmotic Instability: A Preliminary Case Series of Seven Patients

**DOI:** 10.3390/jcm15166217

**Published:** 2026-08-11

**Authors:** Dae-Woong Kim, Eui-Dong Yeo, Ki Jin Jung, Chang Hwa Hong, Sung Hyun Lee, Jae Young Ji, Dhong-Won Lee, Woo-Jong Kim

**Affiliations:** 1 Department of Orthopaedic Surgery, Soonchunhyang University Cheonan Hospital, Cheonan 31151, Republic of Korea; kkkdw7777@naver.com (D.-W.K.); c89546@schmc.ac.kr (K.J.J.); chhong@schmc.ac.kr (C.H.H.); 2 Department of Orthopaedic Surgery, Veterans Health Service Medical Center, Seoul 05368, Republic of Korea; angel_doctor@naver.com; 3 Department of Orthopaedic Surgery, CHA Bundang Medical Center, CHA University School of Medicine, Seongnam 13449, Republic of Korea; kensin06@hanmail.net; 4 Department of Anesthesiology and Pain Medicine, Soonchunhyang University Cheonan Hospital, Cheonan 31151, Republic of Korea; phmjjy@naver.com; 5 Department of Orthopaedic Surgery, Konkuk University Medical Center, Seoul 05030, Republic of Korea; osdoctorknee@kuh.ac.kr

**Keywords:** ankle fracture, syndesmotic injury, suture tape augmentation, anterior inferior tibiofibular ligament, posterior inferior tibiofibular ligament, syndesmotic reduction

## Abstract

**Background:** In high-grade syndesmotic injuries involving combined disruption of the anterior inferior tibiofibular ligament (AITFL) and posterior inferior tibiofibular ligament (PITFL), most suture-tape augmentation techniques have focused on the AITFL alone. This study evaluated the clinical and radiologic outcomes of a suture-tape-only anterior and posterior (A-P) augmentation technique, with a primary focus on technical feasibility and maintenance of syndesmotic reduction. **Methods:** We retrospectively reviewed consecutive patients who underwent A-P syndesmotic augmentation using nonabsorbable suture tape, based on clinical and radiologic data available from January 2022 through April 2026. Complete disruption of both the AITFL and PITFL was confirmed arthroscopically and by direct visualization. Clinical outcomes were assessed using the Olerud–Molander Ankle Score (OMAS) and visual analog scale (VAS) preoperatively and at the 1-year postoperative assessment. Syndesmotic reduction was evaluated on bilateral computed tomography (CT) scans obtained 1 year postoperatively using the direct anterior difference (DAD), direct posterior difference (DPD), fibular translation (FT), and fibular rotation (FR), with the uninjured contralateral ankle serving as the reference. **Results:** Seven patients (mean age, 35.1 years; mean follow-up, 26.3 months) were included. At the 1-year postoperative assessment, the mean OMAS increased from 1.4 ± 3.8 to 86.4 ± 5.6, whereas the mean VAS score decreased from 8.1 ± 0.7 to 0.3 ± 0.5 (both *p* = 0.016), with preoperative scores obtained in the acute injury setting. For both observers, no individual absolute side-to-side difference exceeded the predefined malreduction threshold for any of the four CT parameters. Bony union was achieved in all cases. One patient developed a superficial wound complication, and no reoperations were required. **Conclusions:** In this small, selected case series, A-P suture-tape augmentation was technically feasible, and syndesmotic reduction was maintained at 1 year postoperatively in patients with combined AITFL and PITFL injuries. These preliminary findings warrant further evaluation in larger comparative studies.

## 1. Introduction

Syndesmotic injury is common, accompanying approximately 17% of ankle sprains and 20% of ankle fractures [1]. In unstable ankle fractures, anatomic reduction of the distal tibiofibular joint (DTFJ) through restoration of the syndesmosis is essential. Untreated syndesmotic injury or syndesmotic malreduction can alter ankle biomechanics by shifting the center of pressure and changing the rotational relationship between the fibula and talus [2]. These alterations may increase tibiotalar contact pressure, ultimately leading to chronic instability, poor functional outcomes, and posttraumatic osteoarthritis [3,4,5,6,7,8]. The syndesmotic complex stabilizes the DTFJ through three-point fixation of the fibula to the tibia by three major structures: the anterior inferior tibiofibular ligament (AITFL), the interosseous ligament (IOL), and the posterior inferior tibiofibular ligament (PITFL) [9].

Although trans-syndesmotic screw fixation has long been considered the standard method and remains the most commonly used technique for syndesmotic stabilization in unstable ankle fractures, rigid screw fixation has recognized limitations and complications [10,11,12,13,14]. Screw fixation provides high primary stability and has a lower initial implant cost. However, it has been associated with syndesmotic malreduction, recurrent diastasis after screw removal, implant-related complications, and relatively high reoperation rates [2,15,16,17,18,19]. These limitations have prompted the development of alternative fixation strategies aimed at providing more physiologic stabilization of the syndesmosis.

Suture-button fixation was introduced as a dynamic alternative to rigid screw fixation, providing more physiologic stabilization of the syndesmosis [17]. Owing to its flexible construct, suture-button fixation has been suggested to permit a certain degree of spontaneous correction of mild malreduction during follow-up [19,20]. However, this corrective potential appears limited and may not fully compensate for greater degrees of malreduction [21]. Furthermore, biomechanical models of high-grade syndesmotic injury have shown that suture-button fixation does not consistently restore rotational and sagittal stability under certain loading conditions, and a tendency toward overcompression or overreduction of the DTFJ has been reported [2,22,23,24,25]. These findings do not suggest that suture-button fixation is inferior in routine clinical practice; rather, they provide a rationale for investigating ligament-oriented approaches designed to approximate the native anatomic orientations of the disrupted restraints in selected high-grade injuries with combined ligamentous disruption.

Footprint-based suture-tape augmentation may facilitate anatomic restoration of the syndesmotic ligamentous restraints by anchoring the tape at the native tibial and fibular insertion sites [26,27]. Direct AITFL augmentation may restore the anterior component of the syndesmotic ring and thereby enhance rotational stability [26,28]. Biomechanical studies have demonstrated that open anterior syndesmotic repair augmented with suture tape provides torsional strength comparable to that of trans-syndesmotic screw fixation while maintaining a flexible construct [28].

However, most clinical applications of suture-tape augmentation for syndesmotic injuries have focused on AITFL alone [26,29]. To the best of the authors’ knowledge, no published studies, other than our previous reports, have evaluated simultaneous AITFL and PITFL augmentation using nonabsorbable suture tape or investigated its clinical and radiologic outcomes [30,31].

Therefore, this study aimed to describe a suture-tape-only anterior and posterior (A-P) augmentation technique and to report its preliminary clinical and radiologic findings in patients with unstable syndesmotic injuries involving combined AITFL and PITFL disruption, with a primary focus on technical feasibility and on whether syndesmotic reduction was maintained at 1 year.

## 2. Materials and Methods

This study was approved by the Institutional Review Board (IRB) of Soonchunhyang University Cheonan Hospital, Cheonan, Republic of Korea (IRB No. 2026-05-050-001).

### 2.1. Patient Selection

We retrospectively reviewed consecutive patients who underwent A-P syndesmotic augmentation using nonabsorbable suture tape, based on medical records and imaging studies available from January 2022 through April 2026. Patients were screened according to the predefined inclusion and exclusion criteria. The inclusion criteria were: (1) surgical stabilization of the syndesmosis using A-P suture-tape augmentation for an acute unilateral ankle injury and (2) availability of preoperative and postoperative radiographs and bilateral computed tomography (CT) scans of sufficient quality for radiologic assessment. The exclusion criteria were established to enable reliable comparison with the uninjured contralateral ankle and to minimize confounding factors that could affect clinical or radiologic assessment. These included: (1) age < 18 years; (2) follow-up < 12 months; (3) a history of ankle fracture or syndesmotic injury in either ankle; (4) a history of arthritis in either ankle; (5) an ipsilateral fracture extending to the tibial plafond; (6) a pathologic fracture; (7) an open ankle fracture; and (8) revision surgery.

Injuries were diagnosed using A-P, lateral, and mortise radiographs of both ankles, together with bilateral CT scans with three-dimensional reconstruction. Injuries were classified according to the Lauge-Hansen classification. At our institution, the indication for additional syndesmotic stabilization was determined by residual arthroscopic instability after anatomic fixation of any associated malleolar fractures, rather than by the presence of ligament injury alone. Accordingly, no additional stabilization was performed when the syndesmosis was stable arthroscopically, even if partial ligamentous injury was observed. In unstable cases, direct visualization was used to characterize the osseous or ligamentous injury pattern. Among these cases, A-P suture-tape augmentation was reserved for patients in whom direct visualization confirmed complete disruption of both the AITFL and PITFL.

**Figure 1:**
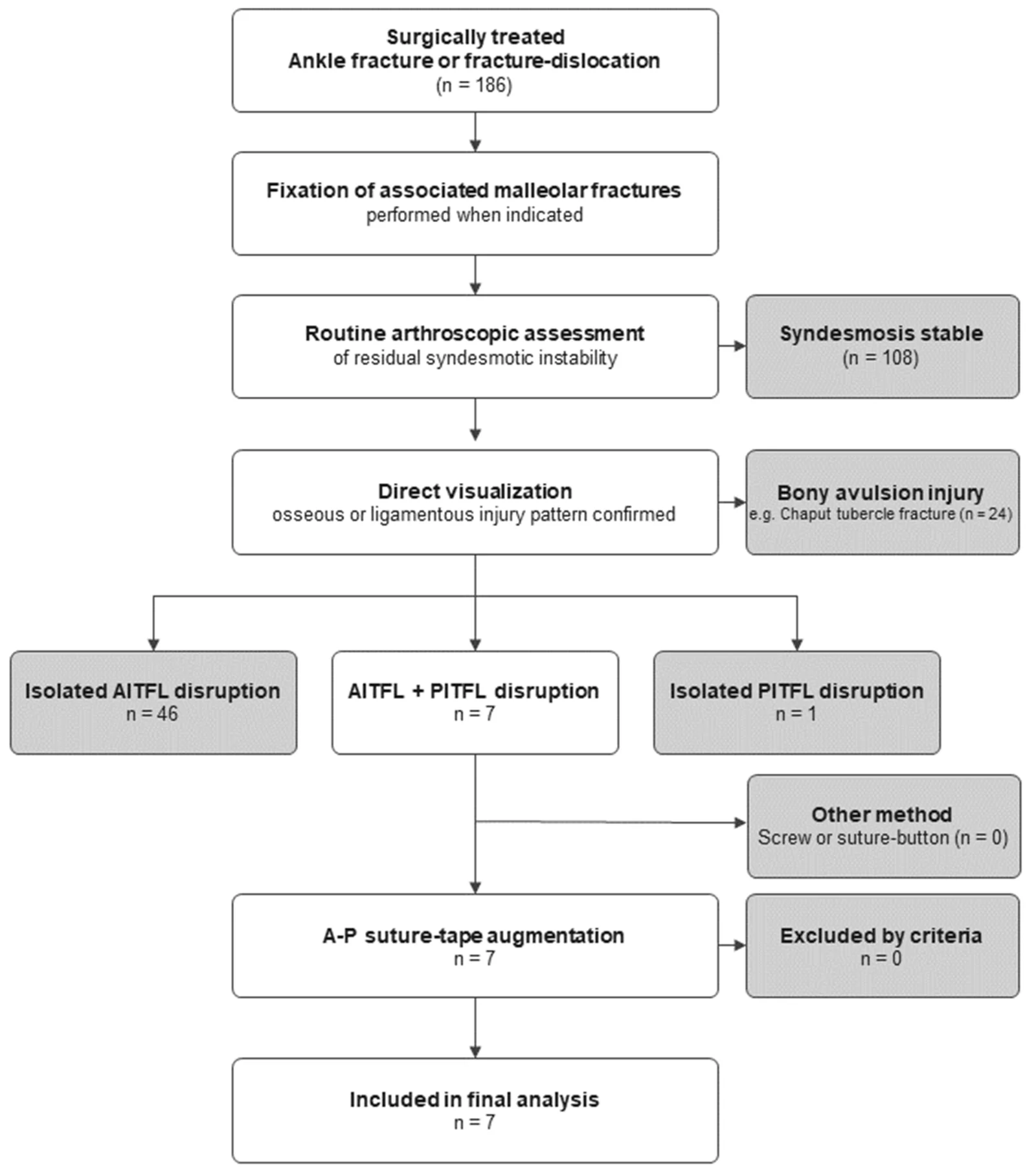
Patient-selection and treatment-decision pathway for anterior and posterior (A-P) suture-tape augmentation. The cohort comprises all patients who underwent surgery for an acute ankle fracture or fracture-dislocation at our institution between January 2022 and April 2026. White boxes indicate the pathway leading to inclusion in the final analysis, whereas shaded boxes indicate patients who did not undergo A-P augmentation or were excluded according to the eligibility criteria. AITFL, anterior inferior tibiofibular ligament; PITFL, posterior inferior tibiofibular ligament.

The patient-selection and treatment-decision pathway is summarized in Figure 1.

### 2.2. Clinical Evaluation

The Olerud-Molander Ankle Score (OMAS) was used to assess overall function and subjective satisfaction following ankle injury [32]. Pain was evaluated using the visual analog scale (VAS). OMAS and VAS scores were recorded preoperatively and at the 1-year postoperative assessment, corresponding to the scheduled 1-year CT follow-up.

### 2.3. Radiologic Evaluation

**Figure 2:**
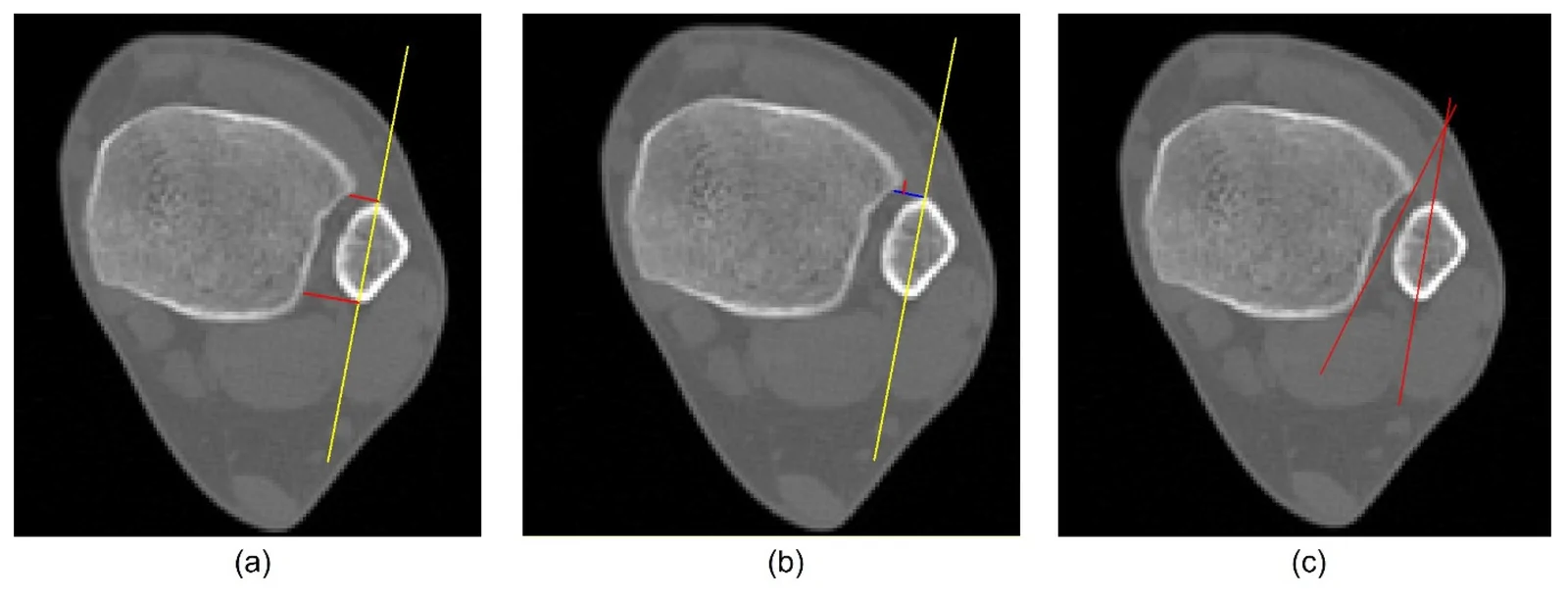
Computed tomography (CT)-based radiologic measurements used in this study. (**a**) Red lines: direct anterior difference (DAD) and direct posterior difference (DPD). Yellow line: fibular orientation line. (**b**) Red line: fibular translation. Yellow line: fibular orientation line. Blue line: DAD reference line, drawn through the anterior end of the fibular orientation line with the same orientation as the DAD measurement line. (**c**) Red line: fibular rotation.

Plain radiographs were obtained at 2, 4, and 6 weeks and 3, 6, and 12 months postoperatively to assess fracture union and overall alignment. Bilateral ankle CT scans were obtained immediately after surgery, at 1 year and annually thereafter. The immediate postoperative scans were used to confirm the initial reduction, whereas quantitative measurements for the present study were performed on the 1-year scans to assess maintenance of syndesmotic reduction. Additional annual CT examinations were obtained with patient consent for clinical surveillance of late changes in syndesmotic alignment and the development of posttraumatic osteoarthritis. Both ankles were imaged in a single acquisition with the patient supine, the knees extended, and both ankles maintained in a neutral position, thereby standardizing limb position and slice orientation between the injured and contralateral sides. Radiologic parameters were measured using the institutional picture archiving and communication system (Dejaview2 version 1.0; Dongeun Information Technology, Bucheon-si, Gyeonggi-do, Republic of Korea). Syndesmotic reduction was evaluated on axial CT images obtained 1 cm proximal to the tibial plafond, where the tibial incisura was most clearly defined (Table 1 and Figure 2) [33]. Four radiologic parameters—direct anterior difference (DAD), direct posterior difference (DPD), fibular translation (FT), and fibular rotation (FR)—were used to assess syndesmotic reduction [34]. Side-to-side differences were calculated using the uninjured contralateral ankle as the reference. Based on previously published bilateral CT criteria, malreduction was defined as an absolute side-to-side difference of >2 mm for DAD, DPD, or FT or >10° for FR [35]. The 2 mm threshold was selected in light of reports linking syndesmotic malreduction to inferior clinical outcomes, including a bilateral CT study using a >2 mm side-to-side criterion [7,36,37]. The 10° threshold exceeds the maximum expected side-to-side rotational variation of 6.5° reported in a cohort of uninjured ankles [38]. Both thresholds were also used to define CT-based malreduction in a multicenter randomized controlled trial [39]. Together, these criteria provide a patient-specific, multiplanar assessment of syndesmotic reduction using thresholds broadly consistent with those applied in previous bilateral CT studies [21,35,39].

All four CT measurements were independently performed for each patient by two observers who were not involved in the surgical procedures and were blinded to the patients’ clinical outcomes and current symptoms. To minimize recall bias and reduce measurement variability, each observer repeated all measurements after a 6-week interval. The mean values of the repeated measurements were used for the final analysis.

### 2.4. Surgical Technique and Rehabilitation

All operations were performed by a single surgeon (W.J.K.). The anesthetic method—general anesthesia, spinal anesthesia, or a lower-extremity peripheral nerve block—was selected according to patient-specific factors, and patient positioning was planned based on the preoperative CT findings. One patient required direct fixation of the posterior malleolus and was, therefore, positioned prone for a posterolateral approach before being repositioned supine to complete the procedure. All other patients remained supine throughout the operation.

Associated malleolar fractures were anatomically reduced and fixed before syndesmotic assessment to ensure that subsequent testing reflected residual ligamentous instability after restoration of the bony anatomy. Lateral malleolar fractures were treated with plate fixation through an anterolateral approach, which also allowed extension to the AITFL footprint when augmentation was required. Medial malleolar fractures were fixed using tension-band wiring or screw fixation through a separate medial approach. In the absence of fractures requiring fixation, ankle arthroscopy was performed at the beginning of the procedure; otherwise, it was performed after fixation of the associated fractures. Syndesmotic stability was assessed arthroscopically, with instability defined as the passage of a 3 mm probe into an abnormally widened tibiofibular joint space. Complete disruption of the PITFL was also identified arthroscopically. Once instability was confirmed, a limited anterolateral incision was made, or the existing incision was extended, to expose the AITFL directly. Complete disruption of the AITFL was confirmed, and its tibial and fibular footprints were identified to guide subsequent anchor placement. The posterior restraint was then addressed. The leg was internally rotated, or the patient was repositioned into the lateral decubitus position when exposure was difficult, and an approximately 5-cm posterolateral incision was made between the lateral malleolus and the Achilles tendon to expose the posterior fibula and the Volkmann tubercle. Complete disruption of the PITFL and its corresponding footprints were identified under direct visualization. With the ankle maintained in a neutral position, the syndesmosis was reduced using a pointed reduction clamp. The clamp was adjusted incrementally while reduction was assessed fluoroscopically and arthroscopically. Arthroscopic probing was used to evaluate the tibiofibular joint space and avoid excessive compression. Once satisfactory reduction had been confirmed, the clamp was maintained during suture-tape fixation.

The augmentation technique was based on a previously reported suture-tape method [31] but combined the anterior and posterior (A-P) restraints into a single construct. Repair was performed using the InternalBrace™ Ligament Augmentation Repair system (Arthrex, Naples, FL, USA), consisting of FiberTape^®^ suture, one 3.5 mm and two 4.75 mm BioComposite™ SwiveLock^®^ anchors (Arthrex, Naples, FL, USA), and the corresponding drills and taps. A 3.4 mm socket was prepared at the tibial AITFL footprint on the Chaput tubercle, a 2.7 mm transfibular tunnel was created from anterior to posterior at the fibular footprint, and another 3.4 mm socket was prepared at the PITFL footprint on the Volkmann tubercle. A pre-twisted passing wire was introduced from posterior to anterior to pass the suture tape through the fibular tunnel, and the tape was secured at the fibular footprint with a 3.5 mm SwiveLock anchor using interference fixation. The posterior limb was then passed beneath the peroneal tendons to the Volkmann tubercle and secured in the 3.4 mm socket with a 4.75 mm SwiveLock anchor while reduction was maintained. Finally, the anterior limb was secured to the Chaput tubercle with the second 4.75 mm SwiveLock anchor, completing the construct. Throughout the procedure, care was taken to position the tape at each ligament footprint and maintain central tunnel placement. Tape tension was applied manually according to the surgeon’s judgment, without the use of a calibrated tensioning device. After completion of the construct, the reduction clamp was released, and reduction was reassessed fluoroscopically and arthroscopically. Arthroscopic probing was repeated to confirm syndesmotic stability before wound closure.

Postoperatively, all patients followed a standardized phased rehabilitation protocol. During the initial protective phase, a short-leg splint was applied for approximately 2 weeks and then replaced with an ankle brace for an additional 4 weeks, with non-weight-bearing maintained during the first 4 weeks. At 4 weeks, gentle passive and active range-of-motion exercises were initiated to minimize stiffness, and weight-bearing was gradually advanced. The brace was discontinued at approximately 6 weeks, by which time a functional range of approximately 15° of dorsiflexion and 35° of plantarflexion was targeted. Between approximately 6 and 12 weeks, rehabilitation focused on restoring strength and dynamic stability. Patients were permitted to walk without the brace, progressive resistance exercises were introduced to strengthen the ankle dorsiflexors, plantarflexors, and surrounding stabilizing muscles, and proprioceptive and balance training, including single-leg standing and similar exercises, was initiated to improve postural control. The target range of motion during this phase was approximately 20° of dorsiflexion and 40° of plantarflexion. Beyond 12 weeks, rehabilitation emphasized functional recovery and a gradual return to preinjury activity levels. Patients with higher physical or athletic demands progressed to sport-specific conditioning, with plyometric and agility training incorporated as needed to restore dynamic motor control. In general, return to routine daily activities was anticipated within approximately 3–4 months after surgery.

### 2.5. Statistical Analysis

All statistical analyses were performed using SPSS software (version 29.0; IBM Corp., Armonk, NY, USA). Continuous variables were summarized as means ± standard deviations, and categorical variables as frequencies and percentages. Given the small sample size, preoperative and 1-year postoperative OMAS and VAS scores were compared using the Wilcoxon signed-rank test. CT findings were analyzed descriptively; individual absolute side-to-side differences were calculated and evaluated against the predefined malreduction thresholds. Intraobserver reliability was assessed separately for each observer by comparing the first and second measurement sessions. Interobserver reliability was assessed using the observer-specific mean of the two sessions. Intraclass correlation coefficients (ICCs) were calculated using a two-way mixed-effects model for absolute agreement based on single measurements, with 95% confidence intervals. All statistical tests were two-sided, and a *p*-value of <0.05 was considered statistically significant.

## 3. Results

### 3.1. Patient Characteristics and Clinical Outcomes

A total of seven patients who underwent A-P syndesmotic augmentation with suture tape were included in this study. During the study period, 186 consecutive patients underwent surgery for an ankle fracture or fracture-dislocation. After fixation of associated malleolar fractures, when indicated, all patients underwent routine intraoperative arthroscopic assessment. No residual syndesmotic instability was identified in 108 patients; this group included patients with partial ligamentous injuries that remained stable arthroscopically and therefore did not require additional syndesmotic stabilization. These injuries were not categorized separately. Among the remaining 78 patients with residual instability, direct visualization identified bony avulsion injuries in 24 patients, isolated AITFL disruption in 46, combined AITFL and PITFL disruption in seven, and isolated PITFL disruption in one. All seven patients with combined disruption underwent A-P suture-tape augmentation; none were treated with trans-syndesmotic screw or suture-button fixation, and none met the exclusion criteria. Accordingly, seven patients were included in the final analysis. Patient, injury, and operative characteristics are presented in Table 2, whereas clinical outcomes and complications are presented in Table 3. The mean age was 35.1 ± 10.5 years, and five patients were male and two were female. According to the Lauge-Hansen classification, three patients had supination–external rotation (SER) type IV injuries and four had pronation–external rotation (PER) type IV injuries. Intraoperative assessment confirmed complete disruption of both the AITFL and PITFL in all seven patients. The mean follow-up duration was 26.3 ± 11.0 months.

At the 1-year postoperative assessment, the mean OMAS increased from 1.4 ± 3.8 preoperatively (range, 0–10) to 86.4 ± 5.6 (range, 80–95), whereas the mean VAS score decreased from 8.1 ± 0.7 (range, 7–9) to 0.3 ± 0.5 (range, 0–1). Both changes were statistically significant according to the Wilcoxon signed-rank test (*p* = 0.016 for both OMAS and VAS); all patients underwent concurrent fixation of associated fractures when indicated (Table 2), and the preoperative scores were obtained in the acute injury setting.

### 3.2. Radiologic Outcomes

Immediate postoperative CT scans confirmed syndesmotic reduction in all patients. Bony union was achieved in all cases by the 1-year follow-up, as confirmed by radiographs and CT.

**Figure 3:**
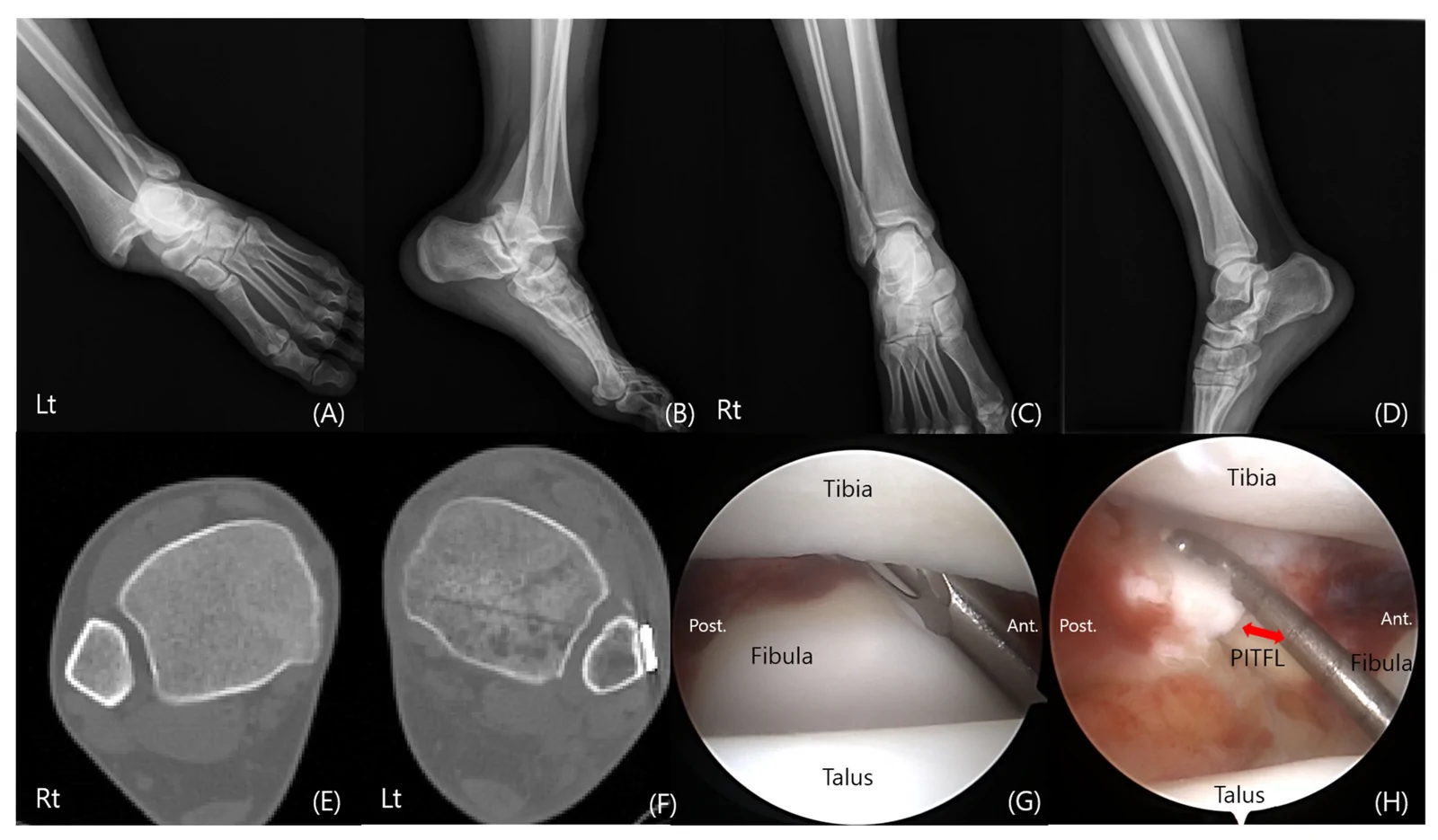
Representative images of Patient 1. (**A**,**B**) Initial radiographs demonstrating a left Lauge-Hansen SER type IV ankle fracture-dislocation with lateral and posterior malleolar fractures: (**A**) anteroposterior view and (**B**) lateral view. (**C**,**D**) Initial radiographs of the uninjured contralateral right ankle: (**C**) anteroposterior view and (**D**) lateral view. (**E**,**F**) One-year postoperative axial CT images obtained 1 cm proximal to the tibial plafond: (**E**) uninjured right ankle and (**F**) injured left ankle. (**G**) Arthroscopic image showing AITFL rupture, with widening of the anterior syndesmotic gap, permitting insertion of a 3 mm shaver tip. (**H**) Arthroscopic image showing PITFL rupture at its tibial insertion. The red arrow indicates the course of the PITFL. SER, supination–external rotation; CT, computed tomography; AITFL, anterior inferior tibiofibular ligament; PITFL, posterior inferior tibiofibular ligament; Lt, left; Rt, right; Ant., anterior; Post., posterior.

Figure 3 presents representative images from Patient 1, who sustained a left Lauge-Hansen SER type IV ankle fracture-dislocation with lateral and posterior malleolar fractures. After fixation of the associated malleolar fractures, arthroscopy and direct visualization confirmed complete disruption of both the AITFL and PITFL, and A-P syndesmotic augmentation was subsequently performed. At 1 year postoperatively, bilateral CT demonstrated maintained distal tibiofibular alignment comparable to that of the uninjured contralateral ankle, and the injured ankle did not meet the predefined CT criteria for malreduction.

The four CT-based parameters were measured on the 1-year postoperative CT scans by two independent observers, using the uninjured contralateral ankle as the reference. The results of the bilateral comparison and malreduction assessment are presented in Table 4 and Table 5, respectively; individual patient-level absolute side-to-side differences are provided in Supplementary Table S1, and the reliability analysis is presented in Table 6.

At the 1-year follow-up, individual absolute side-to-side differences across both observers ranged from 0.12 to 1.56 mm for DAD, 0.12 to 1.25 mm for DPD, 0.04 to 1.26 mm for FT, and 0.50° to 3.80° for FR. Furthermore, no patient met the predefined criteria for malreduction for any parameter.

The radiologic measurements demonstrated excellent reliability. Intraobserver reliability ranged from 0.932 to 0.977 for Observer 1 and from 0.921 to 0.984 for Observer 2, whereas interobserver reliability ranged from 0.927 to 0.960 across the four parameters. The corresponding 95% confidence intervals are presented in Table 6.

### 3.3. Complications

One of the seven patients developed localized superficial wound-edge necrosis at the surgical incision. The wound was managed with local wound care and dressings and healed completely within approximately 4 weeks without debridement or additional surgery. No deep infection, implant-related complication, neurovascular injury, or reoperation occurred in any patient.

## 4. Discussion

This study evaluated the clinical and radiologic outcomes of a suture-tape-only augmentation technique in patients with concomitant AITFL and PITFL injuries. The principal finding from this early clinical experience was that A-P augmentation was technically feasible and that postoperative distal tibiofibular alignment remained within the predefined CT criteria in seven selected patients with high-grade syndesmotic injuries. All patient-level absolute side-to-side differences remained below the corresponding thresholds for both observers. OMAS increased and VAS decreased between the preoperative and 1-year postoperative assessments, and bony union was achieved in all cases. Given the small retrospective case-series design, these findings should be considered preliminary. They are best interpreted as supporting the technical feasibility of this ligament-oriented technique and the maintenance of reduction, rather than as evidence of its clinical effectiveness or of any advantage over screw fixation, suture-button fixation, or AITFL augmentation alone. Larger comparative studies are therefore warranted.

The rationale for addressing both restraints is based on the distinct biomechanical roles of the syndesmotic ligaments, which contribute to maintaining tibiotalar congruence and DTFJ stability against multidirectional forces. The AITFL is a key restraint against external rotation; isolated AITFL injury increases fibular external rotation and posterior translation while reducing resistance to external rotation by approximately 24% [23,40]. Based on these biomechanical findings, several recent studies have advocated anatomic repair or augmentation of the AITFL when external rotational instability is present [2,40]. The PITFL comprises two distinct components: the superficial PITFL (sPITFL) and the deep PITFL (dPITFL). The sPITFL extends obliquely between the posterior aspect of the lateral malleolus and the Volkmann tubercle, whereas the dPITFL forms a compact attachment near the posterior tibial plafond [41,42]. Biomechanical studies have demonstrated that the dPITFL provides approximately 32.7% of the resistance to lateral tibiofibular diastasis [43], whereas sectioning of the sPITFL reduces resistance to internal rotation by 7.8–15.1% and significantly increases sagittal FT [44,45]. These findings highlight the complementary role of the PITFL in maintaining posterior, rotational, and sagittal syndesmotic stability and provide a biomechanical rationale for restoring this ligament in high-grade injuries involving complete PITFL disruption.

Despite its recognized importance, direct PITFL repair using suture tape to reproduce its native anatomic orientation has rarely been reported. To the best of our knowledge, apart from our previous reports [30,31], only a few recent studies have described PITFL repair or reconstruction for posterior lesions, including avulsion-type injuries and complete PITFL rupture [27,46,47]. However, these reports focused exclusively on posterior lesions and did not evaluate simultaneous restoration of the A-P syndesmotic restraints. This distinction may be clinically relevant because syndesmotic injuries are commonly caused by an external rotation mechanism in which the AITFL is typically disrupted first, followed by injury to the IOL and, in more advanced stages, the PITFL. Consistent with this mechanism, isolated PITFL disruption is uncommon and is more frequently accompanied by injury to the AITFL or IOL [48]. This injury pattern is further supported by previous studies demonstrating that PITFL injury was accompanied by AITFL injury in nearly all cases [49,50]. A similar injury pattern was observed during screening of the surgical cohort. Among the 78 patients with residual syndesmotic instability, combined AITFL and PITFL disruption was identified in seven patients, whereas isolated PITFL disruption was identified in only one. Together with previous reports, these findings support consideration of both the anterior and posterior restraints when planning treatment for selected high-grade syndesmotic injuries.

A further challenge in the management of these combined injuries is the risk of postoperative DTFJ malreduction. Previous studies have reported postoperative DTFJ malreduction rates of up to 40% [36,39,51,52,53]. In a bilateral CT-based study using patient-level malreduction criteria that included 2 mm linear and 10° rotational thresholds, Spindler et al. observed a higher malreduction rate in three-ligament than in two-ligament injuries (28% vs. 11%) [21]. These findings underscore the importance of considering coronal diastasis, sagittal FT, and rotational alignment when evaluating syndesmotic reduction in high-grade or multiligament injuries.

The choice of fixation construct is therefore particularly relevant in this setting. Suture-button fixation is widely used as a flexible alternative to trans-syndesmotic screw fixation. However, biomechanical studies using high-grade syndesmotic injury models have suggested that it may not fully restore rotational and sagittal stability under certain loading conditions [22,23,24]. Clanton et al. further demonstrated that none of the tested constructs—including screw fixation and single- or double-suture-button fixation—completely restored native rotational stability or the three-dimensional tibiofibular relationship, leading the authors to propose that anatomic AITFL or PITFL repair might more closely restore the preinjury state [23]. Stake et al. evaluated a posterior malleolar fracture model with disruption of the AITFL and IOL and found that suture-button-containing constructs exhibited significant medial FT under certain loading conditions, suggesting a tendency toward syndesmotic compression or overreduction [22]. A similar tendency toward medial FT has also been reported previously [25]. Although these biomechanical findings do not establish the clinical inferiority of suture-button fixation, they provide a rationale for investigating ligament-oriented approaches in selected high-grade injuries.

Taken together, these considerations led us to adopt a suture-tape-only A-P augmentation technique derived from the syndesmotic InternalBrace introduced by Regauer et al. [54] and described in our previous reports [30,31]. The technique was intended to approximate the native orientations of the anterior and posterior restraints without relying on either trans-syndesmotic screw fixation or suture-button fixation. Although Regauer et al. demonstrated suture-tape augmentation of both the A-P syndesmosis in a cadaveric model, the posterior restraint in their clinical cases was stabilized with a posteriorly directed suture button or screw rather than suture tape. In contrast, the present technique augments both restraints exclusively with suture tape. Whether this construct improves multiplanar stability or reduces the risk of overcompression was not evaluated in the present study.

A distinctive feature of the present technique is the combined use of arthroscopic assessment and direct visualization. Rushing reported that direct visualization was the most reliable intraoperative method for detecting syndesmotic injury and that arthroscopy reliably identified both two- and three-ligament syndesmotic injuries [55]. In addition, several studies have demonstrated that direct visualization during syndesmotic reduction is associated with lower rates of syndesmotic malreduction than indirect reduction methods, with rates of 18% versus 52% reported by Miller et al. and 15% versus 44% reported by Sagi et al. [7,56]. In the present series, arthroscopy was used to identify residual syndesmotic instability and complete PITFL disruption, as well as to confirm syndesmotic stability after fixation. Direct visualization was used to identify surgical landmarks, reconfirm complete disruption of both the AITFL and PITFL, and locate their native insertion sites. On the basis of these reports, direct identification of the injured ligaments and their anatomic footprints was incorporated as a key component of the present technique to reduce reliance on indirect landmarks and facilitate footprint-specific placement of the suture tape along the intended ligament orientations.

The present study used a structured, multiplanar CT-based assessment of syndesmotic reduction. Initial reduction was confirmed on immediate postoperative CT scans, and maintenance of reduction was quantitatively evaluated on CT scans obtained 12 months postoperatively using standardized measurements at a fixed axial level, with the uninjured contralateral ankle serving as the reference. Rather than relying on a single radiologic parameter, we evaluated DAD and DPD to assess coronal-plane displacement (diastasis) of the fibula within the tibial incisura, FT to assess sagittal translation, and FR to assess rotational alignment. The combined use of these four parameters enabled assessment of both the translational and rotational components of syndesmotic reduction. Importantly, postoperative reduction was assessed using individual absolute side-to-side differences and predefined malreduction thresholds, allowing the magnitude of residual asymmetry to be interpreted directly at the patient level. All individual absolute side-to-side differences remained below the predefined thresholds for both observers; the maximum observed difference was 1.56 mm across the three linear parameters and 3.80° for FR. The ICC point estimates and their corresponding 95% confidence intervals further support the reproducibility of the CT measurement protocol.

All seven patients in the present series were included in our previous technical note [31], and one of these patients was also described in an earlier case report [30]. The technical note primarily focused on the surgical technique and rehabilitation protocol and presented patient-level demographic and clinical outcomes in a single descriptive table, without a standardized quantitative CT analysis. In contrast, the present study provides an expanded analysis of the same cohort, including updated clinical outcomes with a minimum follow-up of 12 months, detailed injury and operative characteristics, bilateral CT-based assessment of multiplanar syndesmotic reduction, threshold-based evaluation of malreduction, and intra- and interobserver reliability analyses. A more detailed review of complications also identified one superficial wound complication that had not been reported previously. Accordingly, the present study represents an expanded analysis of the previously reported cohort rather than an independent patient series.

Several limitations should be considered. First, this was a retrospective single-surgeon case series involving only seven patients. However, these patients represented all consecutive cases that met the predefined indication among 186 surgically treated ankle fractures or fracture-dislocations during the study period. Complete disruption of both the AITFL and PITFL was identified in only 7 of 186 cases (3.8%), compared with 46 cases of isolated AITFL disruption. Thus, the small cohort reflects the low frequency of this narrowly defined indication within the screened surgical population; nevertheless, statistical precision and generalizability remain limited. Accordingly, the absence of malreduction in this series should be interpreted descriptively and should not be considered an estimate of the population-level malreduction rate. In addition, clamp compression and tape tension were adjusted according to the surgeon’s judgment rather than quantified using calibrated devices, introducing potential operator-dependent variability; the optimal and reproducible fixation tension remains to be established. Second, because there was no comparison group treated with conventional syndesmotic fixation, no conclusions can be drawn regarding the comparative effectiveness or superiority of the present technique. Moreover, because all patients underwent simultaneous treatment for associated ankle injuries, the observed clinical improvement cannot be attributed specifically to A-P augmentation. In addition, the preoperative OMAS and VAS scores were obtained in the acute injury setting, in which pain and the inability to bear weight resulted in near-floor functional scores and high pain scores. Consequently, the marked preoperative-to-postoperative improvement largely reflects recovery from the acute injury and concurrent treatment rather than the specific effect of A-P augmentation, and this comparison should therefore be interpreted with caution. Third, the follow-up duration was insufficient to assess the long-term maintenance of syndesmotic reduction, the development of posttraumatic osteoarthritis, delayed implant-related complications, or late functional deterioration. Furthermore, radiologic evaluation was based on static postoperative CT measurements, precluding assessment of dynamic or weight-bearing syndesmotic behavior. The radiologic assessment also relied on the uninjured contralateral ankle as the reference, assuming side-to-side symmetry and potentially not accounting fully for normal anatomic variation between ankles. Fourth, biomechanical validation was not performed. Therefore, the ability of the construct to reproduce the intended A-P restraint vectors and restore physiologic syndesmotic stability under rotational and translational loading remains to be determined. Larger comparative studies with longer follow-up, dynamic imaging, and biomechanical testing are needed to establish the clinical effectiveness, mechanical behavior, reproducibility, and safety of this technique. Finally, the observed wound complication rate of 14.3% was based on a single event in this seven-patient cohort and should therefore be interpreted cautiously rather than as an estimate of the population-level wound complication rate.

## Figures and Tables

**Table 1:** Definitions of CT-based radiologic parameters used in this study.

Parameter	Description
Direct anterior difference, mm	Distance between incisura and the anterior end of the fibular orientation line, measured perpendicularly.
Direct posterior difference, mm	Distance between incisura and the posterior end of the fibular orientation line, measured perpendicularly.
Fibular translation, mm	Perpendicular distance between the anterior edge of tibial incisura and the DAD reference line, drawn through the anterior end of the fibular orientation line using the same orientation as the direct anterior difference measurement line.
Fibular rotation, °	The angle between a line connecting the anterior and posterior borders of tibial incisura and a line on the fibula representing its orientation. The angle was considered positive when the fibula was rotated internally relative to the incisura.

**Table 2:** Patient, injury, and operative characteristics.

Patient No.	Age (Years)	Sex	Injured Side	Lauge-Hansen	Fracture Pattern	Fracture Fixation	Additional Procedures	Injury-to- Surgery (Days)
1	23	M	Left	SER IV	Lateral and posterior malleolar	Lateral malleolar plate fixation	External fixation, deltoid ligament repair	7
2	46	M	Right	PER IV	Proximal fibula and posterior malleolar (Maisonneuve)	None	Deltoid ligament repair	1
3	36	M	Right	PER IV	Trimalleolar	Lateral malleolar plate fixation; medial malleolar tension band wiring	External fixation	6
4	37	F	Left	PER IV	Trimalleolar	Lateral and medial malleolar plate fixation	None	1
5	46	M	Left	SER IV	Posterior malleolar	Posterior malleolar screw fixation	Deltoid ligament repair	2
6	39	M	Right	PER IV	Fibular shaft and posterior malleolar (Maisonneuve)	None	None	2
7	19	F	Left	SER IV	Lateral malleolar	Lateral malleolar plate fixation	None	1
Mean ± standard deviation	35.1 ± 10.5	NA	NA	NA	NA	NA	NA	NA

Data are presented as individual values and mean ± standard deviation, where applicable. Fracture fixation refers to fixation of associated fractures and does not include the A-P syndesmotic augmentation performed in all patients. Additional procedures indicate procedures performed during the index surgical episode in addition to fracture fixation and A-P augmentation. Injury-to-surgery was defined as the interval from injury to definitive surgery. SER, supination–external rotation; PER, pronation–external rotation; M, male; F, female; NA, not applicable.

**Table 3:** Clinical outcomes at 1 year and complications during follow-up.

Patient No.	Follow-Up (Months)	OMAS		VAS		Complication
		Pre	1 Year	Pre	1 Year	
1	24	10	80	8	1	None
2	28	0	85	7	0	Superficial wound complication
3	32	0	90	8	0	None
4	12	0	95	8	0	None
5	40	0	80	9	1	None
6	12	0	90	8	0	None
7	36	0	85	9	0	None
Mean ± standard deviation	26.3 ± 11.0	1.4 ± 3.8	86.4 ± 5.6	8.1 ± 0.7	0.3 ± 0.5	NA
*p*-value	NA	0.016		0.016		NA

Data are presented as individual values and mean ± standard deviation, unless otherwise indicated. Preoperative OMAS and VAS scores were obtained in the acute injury setting before surgery, whereas postoperative scores were obtained at the 1-year postoperative assessment. P-values represent paired preoperative-to-postoperative comparisons and were calculated using the Wilcoxon signed-rank test. Complications were recorded throughout the postoperative follow-up period. OMAS, Olerud–Molander Ankle Score; VAS, visual analog scale; Pre, preoperative; NA, not applicable.

**Table 4:** Bilateral comparison of radiologic parameters between the injured and contralateral sides on 1-year postoperative CT scans.

Parameter	Observer 1			Observer 2		
	Uninjured (*n* = 7)	Injured (*n* = 7)	Absolute Side-to-Side Difference	Uninjured (*n* = 7)	Injured (*n* = 7)	Absolute Side-to-Side Difference
DAD, mm	5.40 ± 0.94	5.67 ± 1.05	0.59 ± 0.47 (0.12–1.56)	5.37 ± 0.92	5.77 ± 1.10	0.89 ± 0.29 (0.49–1.29)
DPD, mm	9.96 ± 1.77	10.15 ± 1.58	0.77 ± 0.50 (0.12–1.25)	9.79 ± 1.50	10.10 ± 1.74	0.65 ± 0.37 (0.13–1.05)
FT, mm	1.87 ± 0.85	1.68 ± 0.83	0.54 ± 0.47 (0.04–1.26)	1.78 ± 0.86	1.75 ± 1.00	0.53 ± 0.30 (0.08–0.94)
FR, °	13.50 ± 4.42	14.19 ± 5.01	1.95 ± 1.33 (0.50–3.80)	13.84 ± 5.65	14.46 ± 6.13	1.78 ± 0.81 (0.65–3.00)

Data are presented as mean ± standard deviation. Absolute side-to-side differences are presented as mean ± standard deviation (range) and were calculated for each patient as the absolute difference between the injured and uninjured contralateral ankles. Individual patient-level absolute side-to-side differences are provided in Supplementary Table S1. For each observer, the mean of the two measurements obtained at a 6-week interval was used for analysis. DAD, direct anterior difference; DPD, direct posterior difference; FT, fibular translation; FR, fibular rotation. Clinically, DAD and DPD reflect coronal-plane fibular displacement, FT reflects sagittal translation, and FR reflects rotational alignment. Together, these parameters characterize multiplanar syndesmotic reduction.

**Table 5:** Malreduction rate based on the side-to-side difference criteria.

Parameter	Threshold	Observer 1	Observer 2
DAD	>2 mm	0/7 (0%)	0/7 (0%)
DPD	>2 mm	0/7 (0%)	0/7 (0%)
FT	>2 mm	0/7 (0%)	0/7 (0%)
FR	>10°	0/7 (0%)	0/7 (0%)

Data are presented as *n*/N (%). Malreduction was defined as an absolute side-to-side difference of >2 mm for DAD, DPD, or FT, or >10° for FR. DAD, direct anterior difference; DPD, direct posterior difference; FT, fibular translation; FR, fibular rotation. Clinically, DAD and DPD reflect coronal-plane fibular displacement, FT reflects sagittal translation, and FR reflects rotational alignment. Together, these parameters characterize multiplanar syndesmotic reduction.

**Table 6:** Intraobserver and interobserver reliability for radiologic measurements.

Parameter	Intraobserver Reliability		Interobserver, ICC (95% CI)
	Observer 1, ICC (95% CI)	Observer 2, ICC (95% CI)	
DAD	0.942 (0.862–0.963)	0.921 (0.804–0.956)	0.960 (0.874–0.983)
DPD	0.977 (0.934–0.988)	0.984 (0.940–0.993)	0.946 (0.821–0.971)
FT	0.959 (0.884–0.976)	0.960 (0.899–0.979)	0.927 (0.802–0.962)
FR	0.932 (0.828–0.959)	0.941 (0.832–0.973)	0.937 (0.855–0.970)

Intraobserver ICCs were calculated by comparing the first and second measurement sessions for each observer, whereas interobserver ICCs were calculated using the observer-specific mean of the two sessions. ICCs are presented as two-way mixed-effects, absolute-agreement, single-measure estimates with 95% confidence intervals. DAD, direct anterior difference; DPD, direct posterior difference; FT, fibular translation; FR, fibular rotation; ICC, intraclass correlation coefficient. Clinically, DAD and DPD reflect coronal-plane fibular displacement, FT reflects sagittal translation, and FR reflects rotational alignment. Together, these parameters characterize multiplanar syndesmotic reduction.

## Data Availability

The data presented in this study are available on request from the corresponding author. The data are not publicly available due to privacy and ethical restrictions related to patient confidentiality.

## Supplementary Materials

The following supporting information can be downloaded at https://www.mdpi.com/article/10.3390/jcm15166217/s1. Table S1: Individual absolute side-to-side differences in radiologic parameters between the injured and contralateral sides on 1-year postoperative computed tomography (CT) scans.

## Abbreviations

The following abbreviations are used in this manuscript:

CT	Computed tomography
AITFL	Anterior inferior tibiofibular ligament
PITFL	Posterior inferior tibiofibular ligament
A-P	Anterior and posterior
OMAS	Olerud–Molander Ankle Score
VAS	Visual analog scale
DAD	Direct anterior difference
DPD	Direct posterior difference
FT	Fibular translation
FR	Fibular rotation
DTFJ	Distal tibiofibular joint
IOL	Interosseous ligament
ICC	Intraclass correlation coefficient
CI	Confidence interval
SER	Supination–external rotation
PER	Pronation–external rotation
sPITFL	Superficial posterior inferior tibiofibular ligament
dPITFL	Deep posterior inferior tibiofibular ligament
