# Supplementary material for: Clinical and CT-Based Outcomes of Anterior and Posterior Syndesmotic Augmentation Using Nonabsorbable Suture Tape for Acute Syndesmotic Instability: A Preliminary Case Series of Seven Patients
Source: J Clin Med. 2026 Aug 11;15(16):6217. doi: 10.3390/jcm15166217 (PMC13513389; doi:10.3390/jcm15166217)
Supplement: Supplementary file 1 [file jcm-15-06217-s001.zip › jcm-4443301-supplementary.pdf]

Journal of Clinical Medicine

Supplementary Table S1. Individual **absolute** side-to-side differences in radiologic parameters between the injured and contralateral sides on 1-year postoperative computed tomography (CT) scans.

Patient No.	Absolute side-to-side difference							
	DAD, mm		DPD, mm		FT, mm		FR, °	
	Obs 1	Obs 2	Obs 1	Obs 2	Obs 1	Obs 2	Obs 1	Obs 2
1	0.25	0.72	0.12	0.13	0.04	0.94	0.75	1.90
2	0.52	1.02	0.46	0.86	0.15	0.41	3.80	0.65
3	0.12	0.49	0.94	0.54	0.48	0.52	0.50	2.40
4	0.44	0.84	1.23	0.98	0.20	0.08	2.85	1.50
5	1.56	1.29	1.25	1.05	1.05	0.31	0.55	2.00
6	0.74	1.18	1.21	0.18	1.26	0.84	2.60	1.00
7	0.48	0.70	0.20	0.78	0.61	0.59	2.60	3.00
**Mean ± standard deviation**	0.59 ± 0.47	0.89 ± 0.29	0.77 ± 0.50	0.65 ± 0.37	0.54 ± 0.47	0.53 ± 0.30	1.95 ± 1.33	1.78 ± 0.81
**Range**	0.12–1.56	0.49–1.29	0.12–1.25	0.13–1.05	0.04–1.26	0.08–0.94	0.50–3.80	0.65–3.00
**Malreduction threshold**	>2.0	>2.0	>2.0	>2.0	>2.0	>2.0	>10.0	>10.0

Data are presented as individual values, mean ± standard deviation, and range. Side-to-side differences were calculated as the absolute difference between the injured and uninjured contralateral sides for each patient. For each observer, the mean of two repeated measurements obtained at a 6-week interval was used for analysis. Malreduction was defined as a side-to-side difference > 2 mm for DAD, DPD, or FT, or > 10° for FR; no patient exceeded the threshold for any parameter for either observer. DAD, direct anterior difference; DPD, direct posterior difference; FT, fibular translation; FR, fibular rotation; Obs, observer.
